# Uric acid-to-albumin ratio is associated with metabolic syndrome in a large adult cohort

**DOI:** 10.17305/bb.2026.14193

**Published:** 2026-05-07

**Authors:** Nagihan Akkaş

**Affiliations:** 1 Department of Internal Medicine, Medical Park Gebze Hospital, Kocaeli Health and Technology University, Kocaeli, Türkiye; 2 Department of Internal Medicine, Faculty of Medicine, Istinye University, Istanbul, Türkiye

**Keywords:** Uric acid-to-albumin ratio, UAR, metabolic syndrome, cardiometabolic risk, biomarkers

## Abstract

Serum uric acid has been linked to metabolic syndrome (MetS), although the relationship has been inconsistent. The uric acid-to-albumin ratio (UAR) has emerged as a novel composite biomarker that indicates the balance between systemic prooxidant burden and antioxidant capacity. This study aimed to evaluate the independent association between UAR and MetS in a large, real-world adult cohort. This cross-sectional study utilized retrospectively collected data from 4,596 adults (mean age: 38.78 ± 9.78 years; 64.6% female). MetS was defined according to the modified National Cholesterol Education Program – Adult Treatment Panel III (NCEP-ATP III) framework, excluding waist circumference due to significant missing data. Consequently, MetS classification was based on the remaining four criteria: triglycerides, HDL cholesterol, blood pressure, and fasting plasma glucose. UAR was calculated as uric acid (mg/dL) divided by albumin (g/L) and was dichotomized by the cohort median (low vs. high UAR). The prevalence of MetS was 7.0% (*n* ═ 320). Participants diagnosed with MetS were older and predominantly male compared to those without the condition (45.23 ± 9.93 vs. 38.29 ± 9.60 years; 61.2% vs. 33.4%, respectively; both *P <* 0.001). High UAR was more prevalent in the MetS group (70.9% vs. 44.6%, *P <* 0.001). Multivariable analysis revealed that age (OR 1.06, 95% CI 1.05–1.07), sex (female vs. male OR 0.43, 95% CI 0.33–0.56), and UAR group (OR 1.89, 95% CI 1.43–2.49) were independently associated with MetS (all *P <* 0.001). These findings indicate that UAR is significantly associated with MetS and adverse metabolic features, suggesting a relationship with cardiometabolic risk and reflecting an underlying metabolic imbalance.

## Introduction

Metabolic syndrome (MetS) encompasses a cluster of interrelated cardiometabolic risk factors, including abdominal obesity, lipid abnormalities, hypertension, and impaired glucose regulation. Collectively, these factors significantly elevate the risk of cardiovascular disease and mortality. Emerging evidence indicates that serum uric acid (SUA), previously regarded as an inert end product of purine metabolism, may contribute to the pathogenesis of metabolic and cardiovascular disorders, rather than serving solely as a passive biomarker [1, 2]. Numerous epidemiological studies have identified a significant correlation between elevated SUA levels and the prevalence of MetS. In older populations, SUA has been linked to both prevalent and incident MetS, with sex-specific cutoff values determined through receiver operating characteristic analyses [3]. Similarly, cross-sectional studies involving individuals with type 2 diabetes mellitus have shown that elevated SUA concentrations are independently associated with the presence of MetS and its related complications, such as microalbuminuria [4]. Prospective cohort studies further corroborate this relationship, revealing that baseline SUA levels are significantly associated with future MetS development, particularly among women [5, 6]. However, not all investigations consistently support a predictive role for uric acid. In populations with a high prevalence of obesity, SUA did not independently predict incident MetS after adjusting for obesity-related parameters, implying that body composition and metabolic context may affect the observed association [7]. Nonetheless, systematic reviews and meta-analyses involving large cohorts consistently demonstrate that individuals with MetS exhibit higher SUA concentrations compared to controls, reinforcing the validity of this association [8–10]. Beyond epidemiological correlations, hyperuricemia has been mechanistically connected to insulin resistance, oxidative stress, endothelial dysfunction, and low-grade systemic inflammation, all of which are integral to MetS pathophysiology [2, 11]. These findings have sparked interest in composite biomarkers that may more effectively capture the interplay between pro-inflammatory and protective factors within cardiometabolic risk profiles. Recently, the uric acid-to-albumin ratio (UAR) has emerged as a novel biomarker, integrating the potential pro-oxidant effects of uric acid with the anti-inflammatory and antioxidant properties of albumin. UAR has been associated with cardiovascular disease, mortality, and adverse cardiometabolic outcomes across diverse populations [12–14]. Despite the increasing interest in UAR as a prognostic marker in cardiovascular contexts, its relationship with MetS and its individual components remains inadequately defined in large, real-world clinical datasets. Therefore, the present study aims to evaluate the association between UAR and MetS in a substantial cohort of adults. Additionally, we investigate the relationship between UAR and individual metabolic components to elucidate its potential role as a cardiometabolic biomarker.

## Materials and methods

### Ethical approval statement

The research was conducted in accordance with the principles of the Declaration of Helsinki. Ethical clearance was obtained from the Human Research Ethics Committee of Istinye University prior to data analysis. The study was reviewed at the committee meeting held on September 3, 2025 (Meeting No: 2025/08) and received approval (Protocol No: 2025-264). This study utilized a cross-sectional analysis of retrospectively collected data based on existing clinical records. Prior to analysis, all patient data were irreversibly anonymized. Given the retrospective nature of the study and the absence of identifiable personal information, the institutional ethics committee granted a waiver of informed consent.

### Study design and setting

A cross-sectional analysis of retrospectively collected data was conducted to assess the association between UAR and the diagnostic components of MetS using routinely collected clinical data. Data were extracted from the institutional hospital information system and laboratory information system. The analysis was based exclusively on routinely recorded biochemical and clinical parameters, with no direct patient intervention.

### Study population

**Figure 1.**
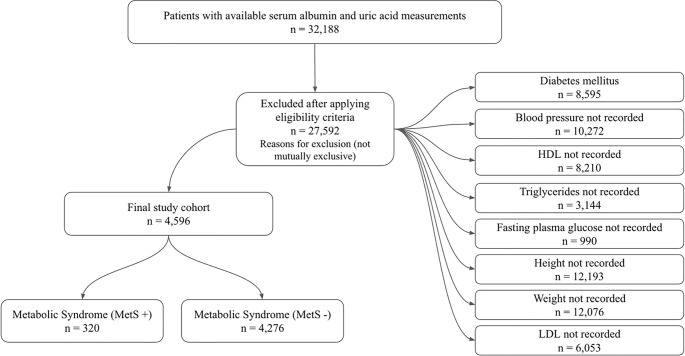
**Flow diagram of the study cohort selection process.** A total of 32,188 patients with available serum albumin and uric acid measurements were screened. Following the application of eligibility criteria, which excluded patients with diabetes mellitus and those missing essential clinical and laboratory variables, 4,596 patients were included in the final analysis. The reasons for exclusion are presented and are not mutually exclusive.

Adult patients (≥18 years) with available same-day SUA and albumin measurements between 2020 and 2025 were screened for eligibility. The study population included both outpatient and inpatient encounters recorded in the hospital information system. These records reflect routine clinical practice and were not restricted to a specific disease group or clinical indication, resulting in a heterogeneous real-world population that may encompass individuals undergoing evaluation for various clinical reasons. A total of 32,188 records were reviewed. The study cohort selection process, including the stepwise application of inclusion and exclusion criteria, is illustrated in Figure 1. The detailed inclusion and exclusion criteria applied to derive the final study cohort are described below.

### Inclusion and exclusion criteria

Patients were eligible for inclusion if they were aged ≥18 years and had available same-day measurements of SUA and albumin between 2020 and 2025. To ensure data completeness and analytical consistency, a complete-case inclusion strategy was implemented. Only patients with complete data for key clinical and laboratory variables—including SUA, albumin, fasting plasma glucose, triglycerides, HDL cholesterol, LDL cholesterol, blood pressure measurements, height, and weight—were included in the final analysis.

As no duplicate entries were present in the dataset, each record corresponded to a unique individual. Patients with a documented diagnosis of diabetes mellitus were excluded to minimize confounding associated with established metabolic disease. Additionally, individuals with missing essential laboratory or clinical data were excluded from the analysis.

After applying all inclusion and exclusion criteria, 4,596 cases were included in the final analysis.

### Definition of MetS

MetS was defined using a modified National Cholesterol Education Program Adult Treatment Panel III (NCEP-ATP III) definition that excluded waist circumference [15]. In accordance with these criteria, the diagnosis was established when at least three of five predefined cardiometabolic components were present. These components include central (abdominal) obesity, defined by a waist circumference of ≥102 cm in men and ≥88 cm in women; elevated triglyceride levels of ≥150 mg/dL; reduced high-density lipoprotein cholesterol concentrations (<40 mg/dL in men and <50 mg/dL in women); increased blood pressure values (systolic ≥130 mmHg and/or diastolic ≥85 mmHg) or ongoing antihypertensive therapy; and impaired fasting plasma glucose levels of ≥100 mg/dL. The requirement for three or more concurrent abnormalities reflects the clustering nature of the metabolic risk factors underlying the syndrome.

Waist circumference measurements were not systematically recorded in the dataset and were available only for a very limited subset of patients (*n* ═ 15; 0.3%). Due to the high degree of missing data and the potential risk of selection bias, waist circumference was excluded from the operational definition of MetS. Consequently, MetS was defined as the presence of at least three abnormal findings among the remaining four NCEP-ATP III components: triglycerides, high-density lipoprotein cholesterol, blood pressure, and fasting plasma glucose. A binary MetS variable (MetS (+) / MetS (-)) was generated based on this definition.

Given the absence of waist circumference data, body mass index (BMI) was retained in the analysis as a descriptive anthropometric variable to characterize general adiposity. However, BMI was not employed as a diagnostic component of MetS and did not replace waist circumference in the definition.

### Missing data

To ensure data completeness and analytical consistency, a complete-case inclusion strategy was applied. Only patients with complete data for all key clinical and laboratory variables were included in the final analysis.

### Exposure definition

The primary exposure variable was the UAR, calculated as SUA (mg/dL) divided by serum albumin (g/L). When multiple paired measurements were available, the earliest eligible measurement was utilized.

### Outcome definition

The main outcome variable was the diagnosis of MetS based on the criteria outlined above. Beyond the primary analysis, limited secondary analyses were performed to descriptively examine the associations between UAR and individual cardiometabolic components, which should be interpreted as exploratory rather than inferential.

### Data preprocessing

Data preprocessing and preparation were conducted using Wanalyzer v3.0 (WisdomEra Corp., Istanbul, Turkey), a Python-based analytical environment that integrates scientific computing libraries (https://wisdomera.io; accessed on 01 April 2026). The analytical workflows employ widely validated open-source statistical libraries, ensuring reproducibility and methodological transparency. This platform is directly integrated with hospital information systems (HIS) and laboratory information systems (LIS), facilitating secure extraction, structured transformation, and standardized processing of routinely collected clinical data. It was utilized for data cleaning, normalization, transformation, feature structuring, and dataset harmonization before analysis. Both parametric and non-parametric preprocessing procedures were applied as appropriate based on data distribution characteristics and variable types.

### Statistical analysis

All statistical analyses were performed using Wistats v3.0 (WisdomEra Corp., Istanbul, Turkey), a Python-based analytical platform that incorporates the SciPy, scikit-learn, and statsmodels libraries (https://wistats.wisdomera.io; accessed on 01 April 2026). These platforms utilize widely validated open-source statistical libraries and do not incorporate proprietary analytical methods. Continuous variables were assessed for normality using the Shapiro–Wilk test. Normally distributed variables are presented as mean ± standard deviation (SD), while non-normally distributed variables are expressed as median and interquartile range (IQR). Categorical variables are reported as frequencies (N) and percentages (%). Comparisons between participants with and without MetS were conducted using the independent samples *t*-test for normally distributed variables and the Mann–Whitney *U* test for non-normally distributed variables. Associations between categorical variables were evaluated using the Chi-square test, with Fisher’s exact test applied when expected cell counts were below five to ensure accurate statistical inference. Correlations between UAR and individual metabolic parameters were assessed using Pearson or Spearman correlation coefficients based on distributional characteristics.

To evaluate the independent association between UAR and MetS, logistic regression models were constructed, with effect estimates reported as odds ratios (ORs) and 95% confidence intervals (CIs). The simultaneous inclusion of MetS diagnostic components (HDL cholesterol group, triglycerides group, fasting plasma glucose group, and blood pressure group) alongside the composite MetS outcome resulted in perfect multicollinearity. As MetS is defined by these variables, their inclusion produced deterministic relationships between predictors and the dependent variable, resulting in a singular design matrix and unstable parameter estimates. To prevent circular modeling and overadjustment bias, these variables were excluded from the final multivariable model.

Additionally, modeling UAR as a continuous variable resulted in quasi-complete separation, characterized by extremely large odds ratios and non-estimable confidence intervals, indicating model instability. To enhance model stability and clinical interpretability, UAR was dichotomized based on the median value of the study population, creating low- and high-UAR groups. This median-based cutoff was utilized for analytical purposes to improve model stability and should not be interpreted as a clinically validated threshold. Therefore, the final multivariable logistic regression model included UAR group adjusted for age and sex. The primary analysis was defined as the multivariable logistic regression model using dichotomized UAR, selected for model stability and clinical interpretability, while secondary analyses were limited and considered exploratory.

All statistical tests were two-sided, and a p-value < 0.05 was deemed statistically significant.

## Results

### Baseline characteristics and demographic overview

A total of 4,596 participants were included in the study, of whom 320 (6.9%) were diagnosed with MetS, while 4,276 (93.1%) did not meet the MetS criteria. The baseline demographic and clinical characteristics of the study cohort are summarized in Table 1. Participants with MetS were significantly older than those without MetS and were more frequently male (both *P <* 0.001). Uric acid levels were significantly higher in the MetS group, and UAR values were elevated compared to those without MetS. As anticipated, variables used in defining MetS differed significantly between groups and are presented in detail in Table 1.

**Table 1:** Baseline demographic and clinical characteristics of the study cohort

	**cases** **Mean ± SD or *n* (%)**
**N**	4596
**Age**	38.78 ± 9.78
**Gender**	
female	2970 (64.6%)
male	1626 (35.4%)
**Uric acid (mg/dl)**	5.07 ± 1.44
**Albumin (g/l)**	45.92 ± 3.10
**Systolic blood pressure (mmHg)**	115.62 ± 14.71
**Diastolic blood pressure (mmHg)**	74.03 ± 9.30
**Height (cm)**	167.10 ± 9.19
**Weight (kg)**	77.58 ± 18.12
**BMI**	27.70 ± 5.74
**HDL cholesterol (mg/dL)**	50.51 ± 13.29
**LDL cholesterol (mg/dL)**	120.63 ± 33.53
**Triglycerides (mg/dL)**	112.51 ± 78.80
**Fasting plasma glucose (mg/dL)**	92.77 ± 8.72
**BMI group**	
normal	1481 (32.2%)
obese	1321 (28.7%)
overweight	1683 (36.6%)
underweight	111 (2.4%)
**UAR**	0.11 ± 0.036
**UAR group**	
high	2133 (46.4%)
low	2463 (53.6%)
**Fasting plasma glucose group**	
high	916 (19.9%)
low	3680 (80.1%)
**Triglycerides group**	
high	951 (20.7%)
low	3645 (79.3%)
**HDL group**	
High	2797 (60.9%)
Low	1799 (39.1%)
**Blood pressure group**	
High	852 (18.5%)
Low	3744 (81.5%)
**Metabolic syndrome**	
no	4276 (93.0%)
yes	320 (7.0%)

Abbreviations: BMI, body mass index; HDL, high-density lipoprotein; LDL, low-density lipoprotein; n, number; SD, standard deviation; UAR, uric acid-to-albumin ratio.

The UAR, the primary biomarker evaluated in this study, was significantly higher in the MetS (+) group (0.12 ± 0.033) compared to the MetS (-) group (0.11 ± 0.029, *P <* 0.001). When participants were categorized according to the median UAR value (0.11), a significantly higher proportion of individuals with MetS were observed in the high-UAR group (70.9%) compared to the low-UAR group (29.1%) (*P <* 0.001).

### Comparative analysis according to MetS status

To evaluate baseline disparities and establish the clinical context for metabolic risk, we compared the demographic, anthropometric, and biochemical characteristics of the 4,596 participants based on their MetS status (Table 2).

**Table 2:** Comparative outcomes between patients with and without metabolic syndrome

	**MetS (--)** **Mean ± SD, N (%)**	**MetS (+)** **Mean ± SD, N (%)**	***P***
**N**	4276 (93%)	320 (7%)	
**Age**	38.29 ± 9.60	45.23 ± 9.93	**<0.001**
**Gender**			**<0.001**
female	2846 (66.6%)	124 (38.8%)	
male	1430 (33.4%)	196 (61.2%)	
**Uric acid (mg/dL)**	5.00 ± 1.40	6.02 ± 1.57	**<0.001**
**Albumin (g/L)**	45.89 ± 3.10	46.31 ± 3.05	**0.011**
**Systolic blood pressure (mmHg)**	114.15 ± 13.25	135.22 ± 18.74	**<0.001**
**Diastolic blood pressure (mmHg)**	73.32 ± 8.75	83.53 ± 11.01	**<0.001**
**Height (cm)**	166.89 ± 9.12	169.93 ± 9.55	**<0.001**
**Weight (kg)**	76.66 ± 17.73	89.80 ± 18.90	**<0.001**
**HDL cholesterol (mg/dL)**	50.61 ± 13.39	49.13 ± 11.94	0.101
**LDL cholesterol (mg/dL)**	119.21 ± 33.01	139.61 ± 34.64	**<0.001**
**Triglycerides (mg/dL)**	107.34 ± 73.77	181.62 ± 106.61	**<0.001**
**Fasting plasma glucose (mg/dL)**	92.01 ± 8.26	102.83 ± 8.41	**<0.001**
**BMI**	27.44 ± 5.62	31.11 ± 6.16	**<0.001**
**UAR**	0.11 ± 0.029	0.13 ± 0.033	**<0.001**
**UAR group**			**<0.001**
high	1906 (44.6%)	227 (70.9%)	
low	2370 (55.4%)	93 (29.1%)	
**Fasting plasma glucose group**			**<0.001**
high	658 (15.4%)	258 (80.6%)	
low	3618 (84.6%)	62 (19.4%)	
**Triglycerides group**			**<0.001**
high	735 (17.2%)	216 (67.5%)	
low	3541 (82.8%)	104 (32.5%)	
**HDL group**			**<0.001**
high	2539 (59.4%)	258 (80.6%)	
low	1737 (40.6%)	62 (19.4%)	
**Blood pressure group**			**<0.001**
high	591 (13.8%)	261 (81.6%)	
low	3685 (86.2%)	59 (18.4%)	

Abbreviations: BMI, body mass index; HDL, high-density lipoprotein; LDL, low-density lipoprotein; MetS, metabolic syndrome; N, number of cases; SD, standard deviation; UAR, uric acid-to-albumin ratio.

**Figure 2.**
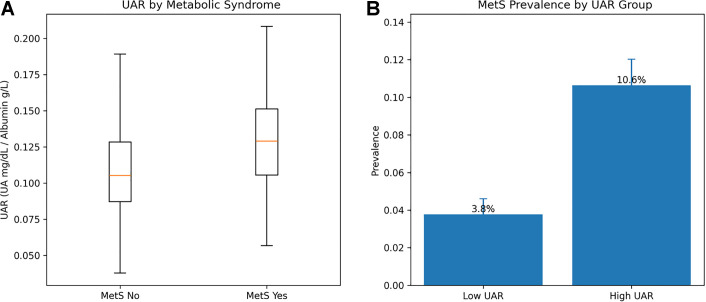
**Association between UAR and MetS.** (A) Distribution of the UAR based on MetS status. Boxplots illustrate the median and interquartile range, while whiskers denote the range excluding outliers. Individuals diagnosed with MetS exhibited significantly higher UAR values compared to those without MetS. (B) Prevalence of metabolic syndrome stratified by UAR group (low vs. high). Bars depict the proportion of individuals meeting MetS criteria within each UAR category, with 95% CIs computed using the Wilson method. The high-UAR group displayed a markedly elevated prevalence of MetS. Abbreviations: CIs, confidence intervals; IQR, interquartile range; MetS, metabolic syndrome; UAR, uric acid-to-albumin ratio.

Participants with MetS were significantly older than those without MetS and were more frequently male (both *P <* 0.001). SUA levels and UAR values were also significantly higher in the MetS (+) group, and the proportion of individuals in the high-UAR group was substantially greater among those with MetS (70.9% vs. 44.6%, *P <* 0.001). As expected, variables used in defining MetS also differed significantly between groups and are presented in detail in Table 2. Figure 2 summarizes the distribution of UAR according to MetS status.

### Clinical stratification by UAR groups

To evaluate the metabolic profile and clinical characteristics across different risk strata, the comparative outcomes of the participants according to their UAR groups (low vs. high) are detailed in Table 3.

**Table 3:** Comparative outcomes based on UAR groups: Low versus high

	**high** **Mean ± SD, N (%)**	**low** **Mean ± SD, N (%)**	***P***
**N**	2133 (46.4%)	2463 (53.6%)	
**Age**	39.81 ± 10.33	37.88 ± 9.19	**<0.001**
**Gender**			**<0.001**
female	875 (41.0%)	2095 (85.1%)	
male	1258 (59.0%)	368 (14.9%)	
**BMI**	29.70 ± 5.72	25.96 ± 5.16	**<0.001**
**Fasting plasma glucose group**			**<0.001**
high	524 (24.6%)	392 (15.9%)	
low	1609 (75.4%)	2071 (84.1%)	
**Triglycerides group**			**<0.001**
high	666 (31.2%)	285 (11.6%)	
low	1467 (68.8%)	2178 (88.4%)	
**HDL group**			**<0.001**
high	1163 (54.5%)	1634 (66.3%)	
low	970 (45.5%)	829 (33.7%)	
**Blood pressure group**			**<0.001**
High	549 (25.7%)	303 (12.3%)	
Low	1584 (74.3%)	2160 (87.7%)	
**Metabolic syndrome**			**<0.001**
no	1906 (89.4%)	2370 (96.2%)	
yes	227 (10.6%)	93 (3.8%)	

Abbreviations: BMI, body mass index; HDL, high-density lipoprotein; N, number of cases; SD, standard deviation; UAR, uric acid-to-albumin ratio.

When participants were stratified according to the median UAR threshold of 0.11, individuals in the high-UAR group (≥0.11) exhibited a significantly more adverse metabolic profile compared to those in the low-UAR group. Participants with a high UAR were significantly older (39.81 ± 10.33 vs. 37.88 ± 9.19 years), and the group showed a markedly higher frequency of male sex (59.0% vs. 14.9%) compared to the low-UAR group (both *P* < 0.001). Regarding anthropometric measurements, the high-UAR group demonstrated a significantly higher BMI (29.70 ± 5.72 vs. 25.96 ± 5.16 kg/m^2^, *P* < 0.001). Furthermore, the prevalence of individual MetS components was consistently higher in the high-UAR cohort; specifically, elevated fasting plasma glucose (24.6% vs. 15.9%), high triglycerides (31.2% vs. 11.6%), and elevated blood pressure (25.7% vs. 12.3%) were all significantly more frequent (all *P* < 0.001). Reduced HDL cholesterol levels were also more common in the high-UAR group (45.5% vs. 33.7%, *P* < 0.001). Notably, the overall prevalence of MetS was nearly three times higher in individuals with a high UAR compared to those with a low UAR (10.6% vs. 3.8%, *P* < 0.001). These findings suggest that UAR is associated with systemic metabolic dysfunction across all measured parameters.

### Logistic regression analysis

To evaluate the independent association between UAR and MetS, a multivariable logistic regression model was constructed, including age, sex, and UAR group (Table 4). Variables that constitute the definition of MetS (HDL cholesterol, triglycerides, fasting plasma glucose, and blood pressure) were excluded from the model to avoid circular interpretation and multicollinearity.

In the multivariable analysis, age was independently associated with MetS (OR 1.06, 95% CI 1.05–1.07, *P <* 0.001). Female sex was associated with lower odds of MetS compared with male sex (OR 0.43, 95% CI 0.33–0.56, *P <* 0.001). The high UAR group was independently associated with MetS compared to the low-UAR group (OR 1.89, 95% CI 1.43–2.49, *P <* 0.001).

### Correlation analysis

**Figure 3.**
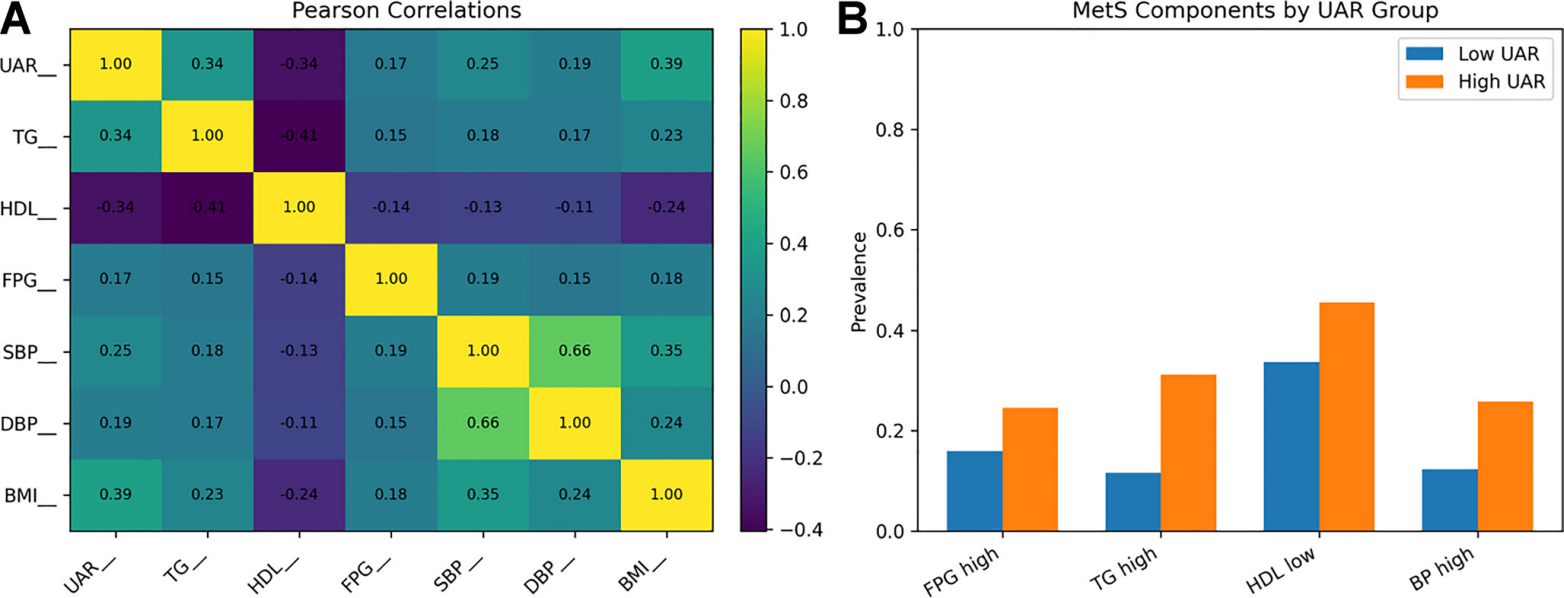
**Relationship between UAR and individual metabolic components.** (A) This Pearson correlation matrix depicts the associations between the UAR and various continuous metabolic parameters, including TG, HDL cholesterol, FPG, SBP, DBP, and BMI. Correlation coefficients (r) are provided within each cell. (B) The prevalence of individual metabolic syndrome components is categorized by UAR group (low vs. high). The bars illustrate the proportion of participants who meet the established criteria for elevated fasting plasma glucose, elevated triglycerides, reduced HDL cholesterol, and elevated blood pressure within each UAR category. Individuals classified in the high-UAR group exhibited a higher prevalence of adverse metabolic components. Abbreviations: BMI, body mass index; DBP, diastolic blood pressure; FPG, fasting plasma glucose; HDL, high-density lipoprotein; r, Pearson correlation coefficient; SBP, systolic blood pressure; TG, triglycerides; UAR, uric acid-to-albumin ratio.

Correlation analyses demonstrated significant associations between UAR and multiple cardiometabolic parameters. Given the overlap between these variables and the definition of MetS, these analyses should be interpreted as descriptive. Detailed results are presented in Figure 3.

**Table 4:** Multivariable logistic regression analysis of factors associated with metabolic syndrome

**Variable**	**OR**	**95% CI**	***P* value**
Age	1.06	1.05--1.07	**<0.001**
Female (vs male)	0.43	0.33--0.56	**<0.001**
UAR group	1.89	1.43--2.49	**<0.001**

The outcome variable was the presence of MetS. The multivariable model includes age, sex, and UAR group (high vs. low). Abbreviations: CI, confidence interval; MetS, metabolic syndrome; OR, odds ratio; UAR, uric acid-to-albumin ratio.

## Discussion

The present study investigates the association between UAR and MetS within a large, real-world cohort of 4,596 adults. MetS was identified in 320 individuals (7.0%). Key findings reveal that patients with MetS exhibited significantly higher SUA levels (6.02 ± 1.57 mg/dL vs. 5.00 ± 1.40 mg/dL, *P <* 0.001) and a significantly different distribution of UAR, with a markedly greater proportion classified in the high-UAR group (70.9% vs. 44.6%, *P <* 0.001). In the final multivariable model adjusted for age and sex, the high-UAR group remained significantly associated with MetS (OR 1.89, 95% CI 1.43–2.49, *P <* 0.001), alongside age (OR 1.06, 95% CI 1.05–1.07, *P <* 0.001) and sex (female vs. male OR 0.43, 95% CI 0.33–0.56, *P <* 0.001). These findings suggest that UAR may serve as a readily available biomarker linked to systemic metabolic dysfunction.

The relationship between SUA and MetS has been extensively documented in epidemiological and clinical studies. Numerous cohort and cross-sectional investigations have demonstrated that elevated SUA levels correlate with an increased prevalence of MetS and its components, including dyslipidemia, hypertension, and insulin resistance [1–3]. For instance, data from the Brisighella Heart Study indicated that higher SUA levels predicted incident MetS over long-term follow-up in older adults [3]. Similarly, prospective evidence from a Korean rural cohort showed that individuals with elevated baseline uric acid levels faced a significantly higher risk of developing MetS over time [6]. In our cohort, individuals with MetS were older (45.23 ± 9.93 vs. 38.29 ± 9.60 years) and predominantly male (61.2% vs. 33.4%), aligning with the established age- and sex-related heterogeneity in cardiometabolic risk observed across populations.

Mechanistically, uric acid has been implicated in various biological pathways pertinent to MetS pathophysiology. The correlations observed between UAR and individual metabolic parameters in our analysis, including multiple cardiometabolic indicators, align with this mechanistic framework. Experimental and clinical evidence suggests that hyperuricemia may induce oxidative stress, endothelial dysfunction, chronic low-grade inflammation, and insulin resistance, all contributing to the clustering of cardiometabolic abnormalities [2, 11]. Interestingly, HDL cholesterol distribution did not conform to the anticipated inverse relationship with MetS in this cohort. This may be explained by the nature of MetS, where multiple metabolic abnormalities coexist, potentially diminishing HDL’s distinct contribution in real-world populations. These mechanisms provide biological plausibility for the observed association between uric acid-related biomarkers and metabolic risk. However, prior studies have reported inconsistent findings regarding the predictive role of uric acid alone. In the Strong Heart Study, uric acid was associated with insulin resistance and inflammatory markers but did not independently predict incident MetS after adjusting for obesity-related factors [7]. This potential effect modification is relevant to our cohort, where BMI was significantly higher in the MetS (+) group (31.11 ± 6.16 vs. 27.44 ± 5.62 kg/m^2^) and also elevated among individuals in the high-UAR group (29.70 ± 5.72 vs. 25.96 ± 5.16 kg/m^2^), indicating that UAR closely tracks with broader metabolic risk. This suggests that the metabolic context and interactions with other physiological systems may influence the observed association between uric acid and MetS. Consequently, recent research increasingly emphasizes composite biomarkers that encompass broader metabolic and inflammatory dynamics rather than relying solely on isolated biochemical parameters. In this context, UAR has emerged as a novel biomarker integrating two biologically complementary components. While uric acid reflects oxidative stress and metabolic dysregulation, albumin is recognized as a marker of nutritional status, antioxidant capacity, and systemic inflammation. The combination of these markers into a ratio may thus provide a more comprehensive representation of systemic metabolic imbalance.

A significant contribution of our study is the emphasis on UAR rather than uric acid alone. UAR integrates uric acid—often viewed as a marker of oxidative stress and metabolic dysregulation—with albumin, which is commonly interpreted as indicative of nutritional status and anti-oxidative/anti-inflammatory capacity. Recent studies increasingly highlight the prognostic value of UAR in cardiovascular and metabolic diseases. For example, Chen et al. reported that UAR independently predicted all-cause and cardiovascular mortality among patients with diabetes, underscoring its potential in risk stratification beyond conventional markers [12]. Similarly, large population-based data from the National Health and Nutrition Examination Survey (NHANES) cohort revealed a non-linear association between UAR and mortality, with higher UAR values correlating with an increased risk of adverse outcomes [13]. In patients with diabetes, UAR has been identified as an independent predictor of all-cause and cardiovascular death, adding prognostic value beyond conventional risk factors [14]. Additional cardiovascular research has further underscored the clinical relevance of UAR. Elevated UAR levels have been linked to atherosclerotic burden in patients with type 2 diabetes, including increased carotid plaque formation [16]. In acute coronary settings, UAR has also been associated with no-reflow phenomena, atrial fibrillation following myocardial infarction, and poor coronary collateral circulation, indicating its broader role in vascular pathology. UAR has been evaluated against other inflammation-based markers and found to be independently associated with poor coronary collateral circulation and related coronary phenotypes [17–21]. Furthermore, it has been associated with the risk of repeat revascularization in young patients with acute coronary syndrome [22]. Collectively, these findings support the notion that UAR reflects systemic biological processes related to oxidative stress, inflammation, and cardiometabolic dysfunction. Although these cardiovascular studies examine endpoints distinct from MetS, they collectively affirm that UAR captures systemic risk biology relevant to cardiometabolic pathways. In our cohort, the prevalence of MetS was nearly threefold higher in the high-UAR group (10.6% vs. 3.8%, *P <* 0.001), reinforcing its association with metabolic risk across the cohort.

Nevertheless, several limitations must be acknowledged. First, the cross-sectional nature of the study design precludes causal inference. Second, certain lifestyle-related confounders such as diet, alcohol consumption, physical activity, and medication use were not available in the dataset. Additionally, information on comorbid conditions, socioeconomic status, and markers of renal and liver function was not consistently available and could not be included in the analysis, leading to the potential for residual confounding due to unmeasured variables. Furthermore, the use of a complete-case inclusion strategy may have introduced selection bias and limited the generalizability of the findings, potentially resulting in a cohort enriched for individuals with higher cardiometabolic risk. Third, waist circumference measurements were not systematically recorded and were therefore excluded from the operational definition of MetS. This deviation from the standard diagnostic framework may have affected classification accuracy. Given that waist circumference is a core diagnostic component of the NCEP-ATP III criteria, its absence may have resulted in misclassification, particularly among individuals whose MetS status is primarily driven by central obesity. Consequently, this limitation may have led to an underestimation of MetS prevalence and could have attenuated the observed associations. Therefore, the findings should be interpreted with caution, especially in populations where central obesity is a dominant phenotype. Fourth, diabetes mellitus was excluded to mitigate confounding related to established metabolic disease; while this enhances internal validity for specific interpretations, it may also limit generalizability to populations with a higher baseline cardiometabolic burden. Fifth, modeling challenges arose when UAR was treated as a continuous predictor, resulting in quasi-complete separation that produced unstable estimates; thus, UAR was dichotomized by the cohort median to enhance interpretability and model stability, albeit at the cost of information loss inherent to categorization. This approach may have introduced non-differential misclassification and reduced statistical power.

Future research should aim to validate these findings in prospective multicenter cohorts with systematically recorded anthropometric and lifestyle variables. Advanced analytical approaches, including penalized regression techniques and machine learning models, may further elucidate optimal clinical thresholds for UAR and improve predictive accuracy. Identifying clinically meaningful thresholds would necessitate dedicated validation studies employing appropriate internal and external validation strategies. Moreover, longitudinal studies are required to ascertain whether elevated UAR precedes the development of MetS or reflects established metabolic dysregulation. Future research incorporating renal function parameters and established cardiovascular risk scores may further clarify the clinical positioning of UAR.

Unlike established cardiovascular risk models designed to predict hard cardiovascular outcomes using longitudinal data, UAR in the present study was evaluated as a cross-sectional biomarker associated with MetS. Therefore, UAR should not be construed as a substitute for validated risk prediction tools but may serve as a complementary marker reflecting underlying cardiometabolic and inflammatory burden. Future longitudinal studies are necessary to determine whether UAR can predict incident MetS and cardiovascular outcomes, and to evaluate its incremental value when integrated into established risk prediction models.

## Conclusion

In this extensive cross-sectional real-world cohort, UAR was independently associated with the presence of MetS and an unfavorable cardiometabolic risk profile. Individuals with elevated UAR values demonstrated a significantly higher clustering of metabolic abnormalities, including disturbances in glucose metabolism, dyslipidemia, increased adiposity, and elevated blood pressure. These findings reinforce the notion that UAR may reflect the combined metabolic and inflammatory burden captured by SUA and albumin levels.

Unlike SUA alone, UAR integrates a potentially pro-oxidative and pro-inflammatory marker with a biomarker typically interpreted as reflecting antioxidant capacity, nutritional status, and systemic inflammatory balance. By combining these complementary biological signals, UAR may provide a more comprehensive representation of the underlying cardiometabolic milieu.

From a clinical perspective, the practical advantages of UAR are noteworthy. Both uric acid and albumin are routinely measured in standard biochemical panels, meaning that UAR can be calculated without additional cost or specialized testing. This accessibility renders it particularly suitable for clinical assessment and population-level evaluation of cardiometabolic profiles. Although UAR was significantly associated with MetS, these findings should not be interpreted as evidence of predictive performance, and no causal or temporal inferences can be drawn due to the cross-sectional design. Further prospective and externally validated studies are required to elucidate its role as a biomarker associated with cardiometabolic risk.

While the observational design precludes causal inference, the independent association between UAR and MetS after adjusting for age and sex suggests that UAR may provide incremental information beyond traditional demographic risk factors. Future prospective and multicenter investigations incorporating structured anthropometric data and advanced modeling approaches are needed to clarify causality, determine clinically relevant thresholds, and evaluate whether UAR improves risk prediction beyond established MetS components.

Collectively, these findings suggest that UAR is a readily available biomarker associated with cardiometabolic risk.

## Data Availability

Anonymized study data are available via the Dataset Sharing Platform of Istinye University: https://dataset.istinye.edu.tr/dataset?did=87. Access is granted for research use under the platform’s licensing and data-sharing policies.
